# NLRP3 Deficiency in Hepatocellular Carcinoma Enhances Surveillance of NK-92 through a Modulation of MICA/B

**DOI:** 10.3390/ijms22179285

**Published:** 2021-08-27

**Authors:** Hwan Hee Lee, Dongoh Kim, Joohee Jung, Hyojeung Kang, Hyosun Cho

**Affiliations:** 1 Department of Pharmacy, Duksung Women’s University, Seoul 01369, Korea; oeo3oeo@gmail.com (H.H.L.); joohee@duksung.ac.kr (J.J.); 2 Duksung Innovative Drug Center, Duksung Women’s University, Seoul 01369, Korea; kdo9710@naver.com; 3 Vessel-Organ Interaction Research Center, VOICE (MRC), Cancer Research Institute, College of Pharmacy, Kyungpook National University, Daegu 41566, Korea

**Keywords:** natural killer (NK) cell, hepatocellular carcinoma (HCC), NLRP3, MICA/B, xenograft model

## Abstract

Human hepatocellular carcinoma (HCC) is the most common and even worse at prognosis. The patients with HCC which accompanied by other diseases, such as cirrhosis, can be limited in various treatments, such as chemotherapy, not HCC patients without other diseases. NLRP3 inflammasome plays an important role in the innate immune response, but emerging evidence has indicated that the NLRP3 inflammasome is implicated in all stages of cancer development. Various cells express NLRP3 protein through the autocrine or paracrine signaling in their environment, but NK cells do not. The expanding evidence shows that patients who suffer from liver cancers have a low frequency of natural killer (NK) cells, and the function of these cells is also impaired. Thus, we examined how the expression of NLRP3 in HCC cells affects cancer surveillance by NK cells in a state of a co-culture of both cells. When the expression of NLRP3 in HCC cells was ablated, MICA/B on the surface of HCC cells was upregulated through the lowered expression of matrix metalloproteinase. The expression of MICA on the surface of HCC cells interacted with the NKG2D receptor on NK-92 cells, which led to NK cytotoxicity. Furthermore, in a xenograft mice model, NLRP3 KO HCC cells delayed tumor development and metastasis as well as increased the sensitivity to NK cell cytotoxicity. Taken together, NLRP3 KO in HCC could enhance NK immunosurveillance through an interaction of NKG2D-MICA.

## 1. Introduction

Human hepatocellular carcinoma (HCC) is very common malignancy, ranking fifth in incidence and third in mortality worldwide [1]. HCC is provoked by various factors, such as hepatitis B, hepatitis C, alcoholism, and diabetes, which induce chronic liver damage [2]. HCC patients are lowest survival in proportion as incidence among all solid cancers about five years [3,4]. The treatment of patients with HCC is often limited because of the presence of other liver diseases, such as cirrhosis, and this imposes an unpredictable pathogenetic mechanism [5,6].

Inflammation is important in the innate immune response, as innate immune cells defend against pathogens, such as macrophages, dendritic cells, neutrophils, and natural killer (NK) cells. Recent reports have shown that inflammation is involved in most stages of cancer development, including tumorigenesis, metastases, and immunosurveillance of immune cells [7,8] in the tumor microenvironment. Inflammasomes are cytoplasmic protein complexes that induce activation, maturation, and the production of inflammatory cytokines from immune cells via the recognition of pathogen- or damage-associated molecular patterns (PAMPs or DAMPs) [9,10]. Among them, the NLRP3 inflammasome is the most well-studied. Previously, it has been shown that the function of the NLRP3 inflammasome produces and activates proinflammatory cytokines such as interleukin (IL)–1β and IL-18 [11]. However, recent studies have described that inflammasome is transcribed in an imbalance in cytoplasmic homeostasis and molecular patterns [12,13], thus the inflammasome is implicated in various inflammatory diseases, such as peritonitis, gouty arthritis, and type 2 diabetes as well as cancers [14,15,16,17,18]. These evidences suggest that NLRP3 inflammasome contributes to the development of various diseases.

NK cells are innate lymphocytes that can directly kill cancer or stressed cells. In human liver, nonparenchymal cells are composed of high-frequency NK and NKT cells (55%), except for parenchymal cells (termed *hepatocytes*) [19]. In addition, NK cells can be recruited into the liver [20], and they endow tumor cells with immune surveillance, such as cytokine production and natural cytotoxicity. However, NK cells are present in a low frequency in patients with HCC, and their function is also impaired, such as their cytotoxic ability or production of interferon-γ [21]. These facts indicate that NK cells may play an important role in liver immunity. NK activity is controlled by a balance between activating and inhibitory receptors. NK activation receptors do not express under normal conditions, and when recognizing ligands of a pathogen, they are expressed on the surface of NK cells. Accumulating data have shown that the ligands on HCC cells for NK-activating receptors, including natural cytotoxicity receptor 3 (NCR3; NKp30), natural killer receptor group 2 member D (NKG2D), and DNAM-1 proteins, are expressed at low levels, which is associated with the processes of disease [22,23]. In particular, ligands that interact with the NKG2D receptor of NK cells are shed on the surface of tumor cells by proteases such as matrix metalloproteinases (MMPs) in the tumor microenvironment [24]. MMPs are a group of endopeptidases that are associated with various physiological processes, including cancer development and the escape of the immune response [25,26]. MMPs have biological activity functions, including gene expression, compartmentalization, and hydrolysis of zymogen, which is regulated by endogenous inhibitors or activating enzymes in the tumor microenvironment [27]. Therefore, we expected that factors expressed in HCC may affect NK cell immunosurveillance through the interaction of the NK-activating receptor and ligand in the tumor microenvironment. In this study, to enhance NK cell immunosurveillance in HCC, we conducted a deletion of NLRP3 gene in HCC using the lentiviral CRISPR-cas9 system and, subsequently, identified how NLRP3 KO in HCC affects the cytotoxic ability of NK cells.

## 2. Results

### 2.1. High NK Cytotoxicity against Human HCC Lacking NLRP3

**Figure 1:**
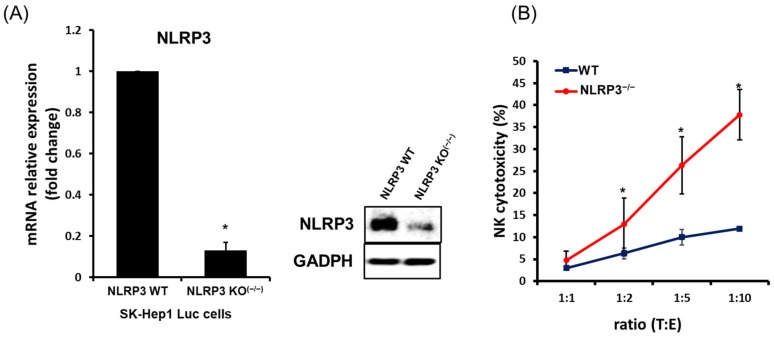
High natural killer (NK) cytotoxicity to human hepatocellular carcinoma (HHC SK-Hep1 Luc cells transfected with Lenti-CRISPR-NLRP3/Cas9. mRNA and protein expression of NLRP3 expression in SK-Hep1 Luc cells transfected with Lenti-CRISPR-Control or NLRP3/Cas9 by qPCR or western blot analysis (**A**). NK cytotoxicity to wild-type SK-Hep1 Luc cells or NLRP3 KO SK-Hep1 Luc cells by LDH-release assay (**B**). All data are compared at * *p* < 0.05. All data were presented as the means ± SD in three independent experiments.

Although NLRP3 is important in innate immune system, natural killer (NK) cells are shown to not express it (Supplementary Figure S1A). Interestingly, we found that the expression of NLRP3 was specific in HCC SK-Hep1 Luc cells (Supplementary Figure S1A and Figure 1A). These results made us raise the question of how the expression of NLRP3 in HCC cells affects NK immune system. Therefore, to identify NK cytotoxicity to human HCC SK-Hep1 Luc cells in the presence or absence of NLRP3, we conducted to a deletion of NLRP3 gene in SK-Hep1 Luc cells using the lentiviral CRISPR-cas9 system. Knockout of NLRP3 in SK-Hep1 Luc cells transfected with lentiviral CRISPR-NLRP3 was confirmed by little expression of mRNA or protein (Figure 1A; *p* < 0.05). Subsequently, we assessed the effect to the function of NK cells in a co-culture with wild-type or NLRP3 KO SK-Hep1 Luc cells. Each of the target cells were treated with NK-92 cells at different ratios (E:T = 1:1, 2:1, 5:1, and 10:1) for 6 h and analyzed by LDH-release assay for identifying NK cytotoxic ability. As shown in Figure 1B, NK cytotoxicity was significantly increased in a co-culture of NLRP3 KO SK-Hep1 Luc cells (at a ratio of 2:1, WT; 6.3%, NLRP3 KO^(−/−)^ 12.9%), and this was increasingly enhanced in a ratio-dependent manner (at a ratio of 5:1; WT 10% to NLRP3 KO^(−/−)^ 26.3%, at a ratio of 10:1; WT 11.9% to NLRP3 KO^(−/−)^ 37.8%). However, the quantity of interferon (IFN)-γ secreted by a co-culture of NK-92 and SK-Hep1 Luc cells was irrelevant to NLRP3 (Supplementary Figure S1B).

### 2.2. High Expression of NK-Activating Receptors in a Co-Culture of NLRP3 KO HCC

**Figure 2:**
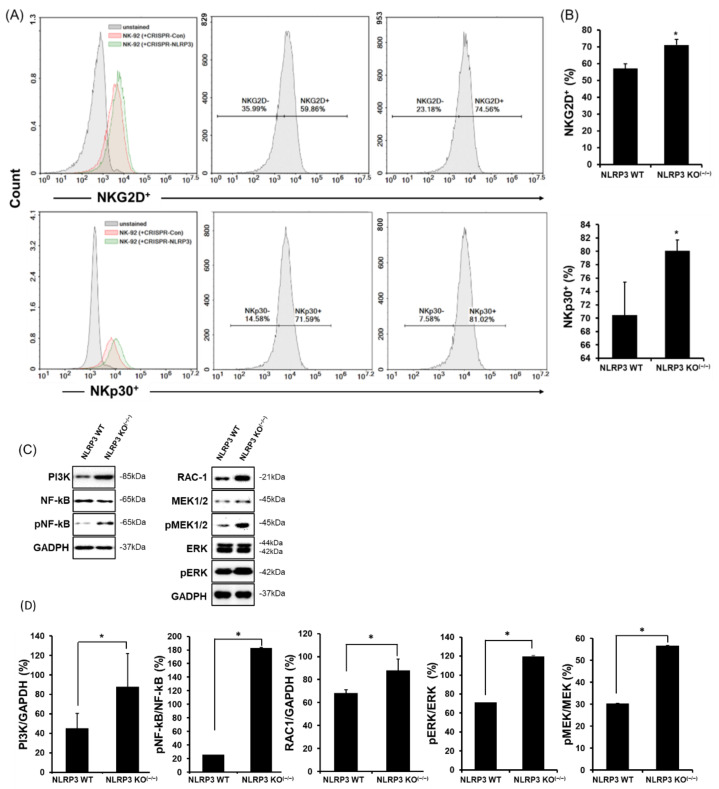
Increases in NK-activating receptors on the surface of NK-92 cells co-cultured with NLRP3 KO HCC. The expression of NKG2D and NKp30 on the surface of NK-92 cells in the co-culture of WT or NLRP3 KO SK-Hep1 Luc cells using flow cytometry (**A**,**B**), and the molecules of the signaling pathway associated with NK-activating receptors in a co-culture of WT or NLRP3 KO SK-Hep1 Luc cells (**C**), and the bands were quantitated as a graph (**D**). * *p* < 0.05. All data are presented as the means ± SD in three independent experiments.

Because NK cytotoxicity is affected by surface receptors, such as activating or inhibitory receptors 4, we thus observed the expression of representative NK-activating receptors, natural killer group 2-member D (NKG2D and natural cytotoxicity receptor (NCRs) which consist of NKp30 and NKp44 in humans. To investigate the expression of NK-activating receptors on NK-92 in a co-culture of SK-Hep1 Luc cells, we co-cultured NK-92 cells with wild-type or NLRP3 KO SK-Hep1 Luc cells, stained the cells with fluorescence antibodies (anti-NKG2D-APC, anti-NKp30-PE, and anti-NKp44-PE) and conducted a flow cytometry analysis. As shown in Figure 2A,B, the positive density of NKG2D and NKp30 on NK-92 cells was higher in NLRP3 KO SK-Hep1 Luc cells as compared with WT (NKG2D^+^; WT 53.79%, NLRP3 KO^(−/−)^ 68.17%, NKp30^+^; WT 71.59%, NLRP3 KO^(−/−)^ 81.02%; *p* < 0.05) however the expression of NKp44 on NK-92 was lower in NLRP3 KO than WT but was not significant. (Supplementary Figure S2A, NKp44^+^; WT 23.01%, NLRP3 KO^(−/−)^ 16.96%; ns). The expression of NK-activating receptors is involved in the recruitment of phosphatidylinositol-3-OH kinase (PI3K), which leads to downstream activation of the MEK/ERK pathway 32. In NK-92 co-cultured with NLRP3 KO SK-Hep1 Luc cells, the expression of PI3K was significantly increased. In addition, an increase in PI3K expression upregulated RAC-1 expression, which induced the phosphorylation of MEK and ERK. These expression of proteins ultimately phosphorylated NF-kB in a nucleus of NK-92 cells (Figure 2C,D, all data; *p* < 0.05).

### 2.3. Increase in MICA/B Expression on the Surface of NLRP3 KO HCC 

**Figure 3:**
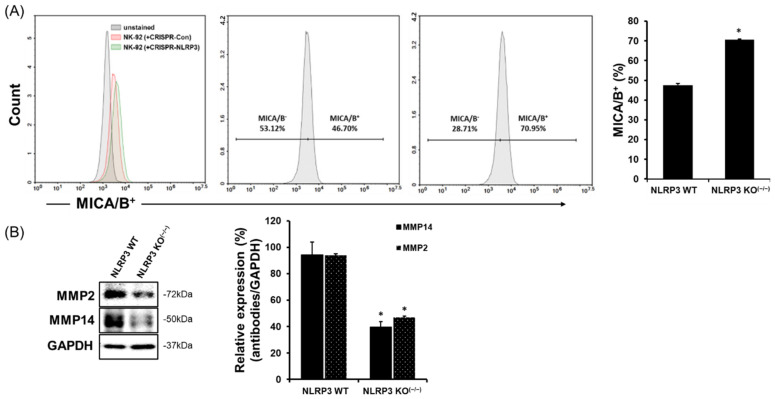
Increases in MICA/B expression on the surface of NLRP3 KO HCC cells through reduced expression of MMP-2 and MMP-14. MICA/B expression on the surface of WT SK-Hep1 Luc cells or NLRP3 KO^(−/−)^ SK-Hep1 using flow cytometry (**A**), the expression of molecules implicated in MICA/B shedding in WT SK-Hep1 Luc cells or NLRP3 KO^(−/−)^ SK-Hep1 Luc cells (**B**). All data are compared at * *p* < 0.05. All data are presented as the means ± SD in three independent experiments.

Our results showed an increased expression of NKG2D and NKp30 on NK-92 cells in a co-culture of NLRP3 KO SK-Hep1 Luc cells (Figure 2A,B). NK-activating receptors interact various ligands on the surface of cancer cells, which results to induce NK cytotoxicity to cancer cells. MICA is a ligand that is expressed in many cancer cells and can bind NKG2D receptor on NK or T cells. In Figure 3A, we found that the positive density of MICA/B was substantially increased on the surface of NLRP3 KO HCC cells (WT 46.70%, NLRP3 KO^(−/−)^ 70.95%; *p* < 0.05) through flow cytometry analysis. We further identified that the activation of MMPs related to MICA/B expression on the surface of cancer cells. In Figure 3B, on the surface of NLRP3 KO HCC cells were achieved through a decreased activation of MMP2 and MMP14 (Figure 3B; *p* < 0.05), however, it could not be distinguished in MMP9.

### 2.4. Suppression of Tumor Development and Metastases, as well as Induction of High Sensitivity to NK Cytotoxicity in Immune-Deficient Mice Developed from NLRP3 KO HCC

**Figure 4:**
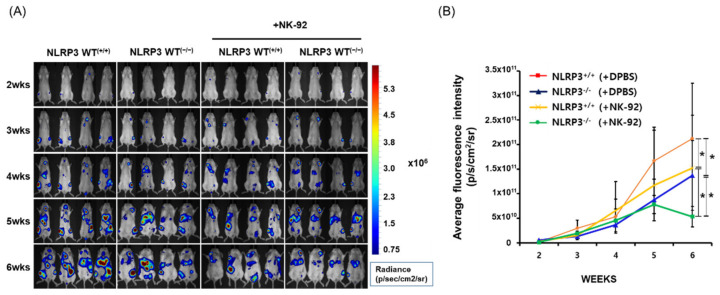
Delay of tumor development and metastasis and improvement in NK cell immunosurveillance through a knockout of NLRP3 in HCC. An in vivo experiment was performed using NRG mice (5 weeks old/female) that lacked lymphocytes (T, B, and NK cells). The tails of mice were intravenously injected with SK-Hep1 Luc cells (1 × 10^6^ cells/mouse) with or without NLRP3 expression. After four days, the mice were treated with an intraperitoneal injection of DPBS or NK-92 cells (2 × 10^6^ cell/mouse) twice a week. Analysis of tumor development and metastases in HCC-implanted mice was conducted using an in vivo imaging system. Fluorescence intensity of luciferase in mice (**A**) and its quantitated graph (**B**). All data are compared at * *p* < 0.05 and presented as the means ± SD in three independent experiments.

To verify whether a deletion of NLRP3 in HCC actually induces effective NK-mediated cancer immune surveillance in vivo, we intravenously injected WT or NLRP3 KO SK-Hep1-Luc cells to NRG mice, restrained in lymphocytes (B, T, and NK cells). Four days after the injection, the mice were treated with DPBS or NK-92 cells via an intraperitoneal injection. There was a significant difference in tumor development and metastasis in mice injected with NLRP3 WT^(+/+)^ SK-Hep1 Luc cells as compared with mice injected with NLRP3 KO^(−/−)^ HCC cells beginning in the fifth week, and this difference increased when the mice were treated with NK-92 cells. In Figure 4A,B, HCC development and metastasis was significantly delayed in mice implanted with NLRP3 KO^(−/−)^ HCC cells compared to mice implanted with NLRP3 WT^(+/+)^ HCC cells (*n* = 6; *p* < 0.05), and this was more when intraperitoneally injected with NK-92 cells (2 × 10^6^ cells/mouse, untreated vs. treated; *p* < 0.05). Interestingly, the sensitivity of NK-92 treatment was greater in mice implanted with NLRP3 KO^(−/−)^ HCC cells as compared with NLRP WT^(+/+)^ HCC cells (*n* = 6; *p* < 0.05).

### 2.5. Increased MICA/B Expression via the Downregulation of MMP2, MMP9, and MMP14 Protein Expression in the Liver Tissue of Mice Implanted with NLRP3 KO HCC

**Figure 5:**
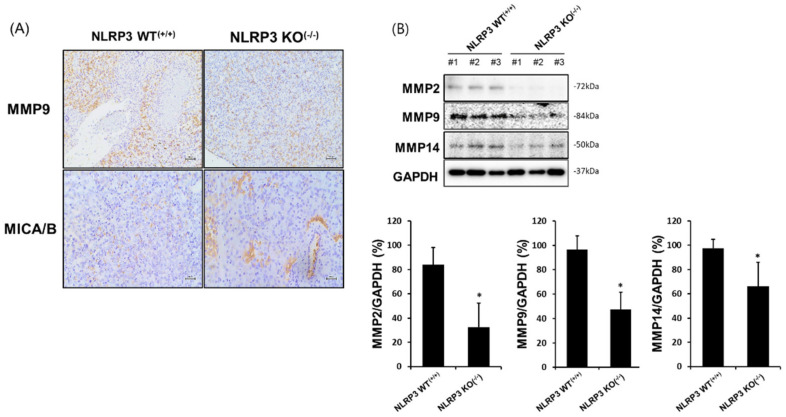

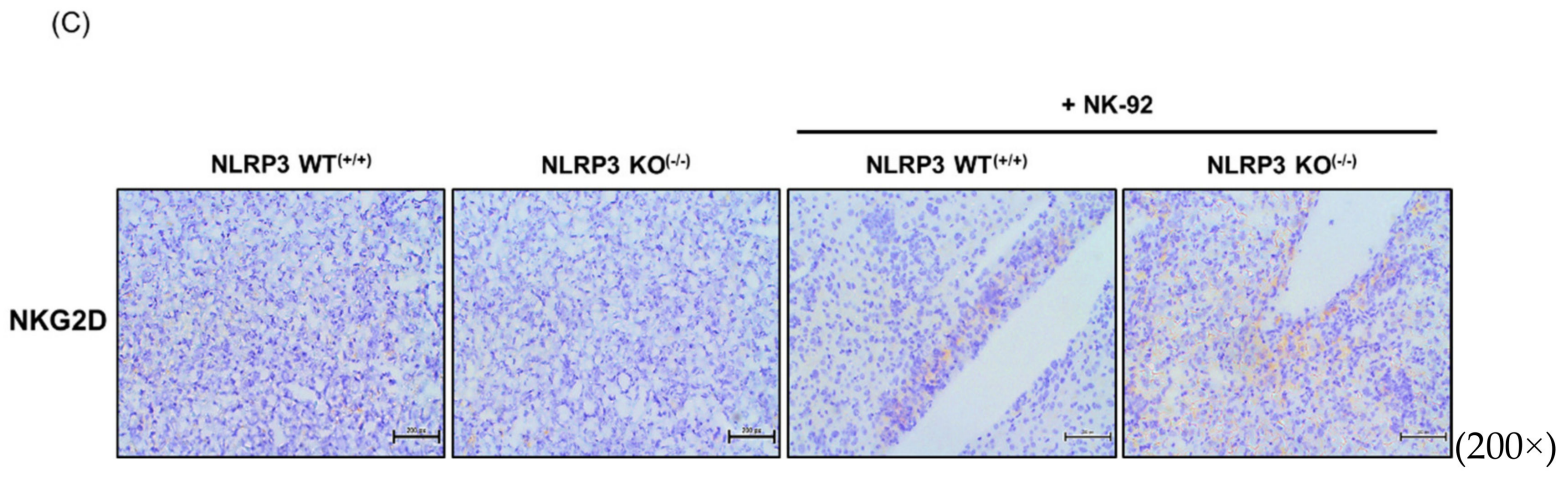
High expression of MICA/B and NKG2D in liver tissue from mice implanted with NLRP3 KO HCC. To examine the expression of MICA/B and NKG2D in liver tissues from mice implanted with WT SK-Hep1 Luc cells or NLRP3 KO SK-Hep1 Luc cells, we used immunohistochemistry staining. Liver tissues of mice that developed HCC were resected and stocked with O.C.T. compound at −80 °C until the experiment. The expression of MICA/B, NKG2D (**A**,**C**), and MMPs (MMP-2, MMP-9, and MMP-14, (**A**,**B**)) in liver tissues from mice implanted with NLRP3 WT^(+/+)^ SK-Hep1 Luc cells or NLRP3 KO^(−/−)^ SK-Hep1 Luc cells in presence or absence of NK-92 treatment (**A**,**B**). Visualization was conducted using light microscopy (100× and 200×). * *p* < 0.05. All data are presented as the means ± SD in three independent experiments.

We found that in vitro experiment, MICA was highly expressed on the surface of HCC SK-Hep1 Luc cells when the NLRP3 was knocked out (Figure 3A). Thus, we expected that MICA could be highly expressed in metastatic liver tissues of mice that developed NLRP3 KO^(−/−)^ SK-Hep1 Luc cells. The observation of MICA expression in liver tissues was confirmed by Western blot analysis and immunohistochemistry (IHC) staining. Interestingly, MICA/B on NLRP3 KO^(−/−)^ HCC metastatic liver tissues was more highly expressed as compared with NLRP WT^(+/+)^ HCC metastatic liver tissues (Figure 5A, lower panel). In addition, MMP-2, MMP-9, and MMP-14 implicated in MICA shedding was significantly decreased in NLRP3 KO^(−/−)^ HCC metastatic liver tissues (Figure 5B; *p* < 0.05). The expression of MMP-9 was difficult to a detection in vitro experiment (Figure 3B; ns) however it could be determined by means of a low expression in NLRP3 KO^(−/−)^ HCC metastatic liver tissues (Figure 5A, upper panel and Figure 5B, *n* = 4; *p* < 0.05). These results coincide with the high expression of NKG2D in NLRP3 KO^(−/−)^ HCC metastatic liver tissues treated with NK-92 cells, as shown in Figure 5C (far right panel).

### 2.6. Expression of Molecules Associated with the Epithelial-to-Mesenchymal Transition and Cancer Development in NLRP3-Lacking HCC Metastatic Liver Tissues

**Figure 6:**
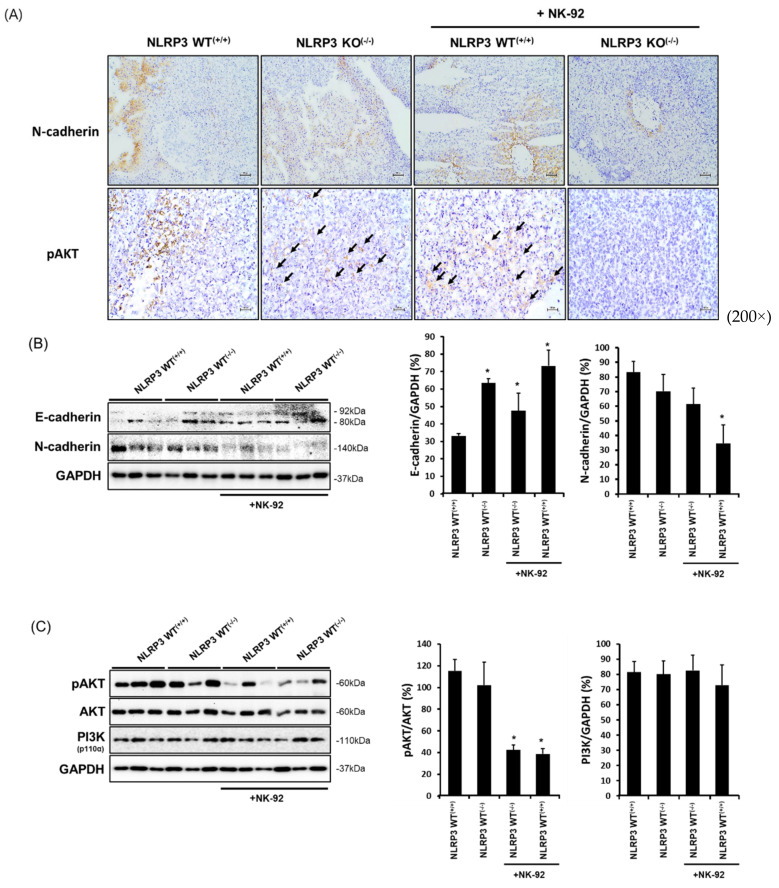
Expression of molecules involved in the epithelial-to-mesenchymal process and cancer development in liver tissues of mice implanted with HCC. The expression of molecules associated with the epithelial-to-mesenchymal transition and cancer development in liver tissues of mice implanted with HCC, immunohistochemistry staining and Western blot analysis. N-cadherin and pAKT in liver tissues from mice implanted with NLRP3 WT^(+/+)^ or NLRP3 KO^(−/−)^ SK-Hep1 Luc cells treated or untreated with NK-92 cells (2 × 10^6^ cells/mouse) by immunohistochemistry (IHC) assay (**A**–**C**), and a graph of the bands. All pictures were visualized by light microscopy (100× and 200×). * *p* < 0.05. All data are presented as the means ± SD in three independent experiments.

The epithelial-mesenchymal transition process is involved in cancer metastasis. Metastatic cancer cells subvert gene expression that is eligible to become a migratory ability 18. Mesenchymal-type cells that can induce metastasis are highly expressed in N-cadherin but have a low expression in E-cadherin. The expression of N-cadherin was significantly decreased in NLRP3 KO^(−/−)^ HCC metastatic liver tissues as compared with NLRP3 WT^(+/+)^ HCC metastatic liver tissues, and this was lowest when NK-92 cells were treated (Figure 6A, upper panel and Figure 6B, *n* = 4; *p* < 0.05). In addition, the phosphorylation of AKT was hardly induced in NLRP3 KO^(−/−)^ HCC metastatic liver tissues treated with NK-92 (Figure 6A, lower panel and Figure 6C, *n* = 4; *p* < 0.05).

## 3. Discussion

Our study found that a deletion of NLRP3 in human HCC SK-Hep1 Luc cells represses cancer development and metastasis of HCC cells as well as enhances the sensitivity of NK cell cytotoxicity in vitro and in vivo. Inflammasomes are protein complexes that contribute to the inflammatory response of innate immune cells, such as macrophages and neutrophils [11,28]. The NLRP3 inflammasome which is one of inflammasomes well-studied has been known to activate pro-inflammatory cytokines, such as IL-1β. However, emerging evidences indicate that the function of NLRP3 inflammasome is associated with many diseases including tumorigenesis [17], autoimmune disorders [14,15], and neurodegenerative diseases [17,18]. According to recent reports, NLRP3 inflammasome is influenced by molecular patterns which caused by an imbalance of cytoplasmic homeostasis [12,13], thus that might be related to cancer development. For example, the NLRP3 inflammasome enhances dendritic cell-mediated activation of T lymphocytes against tumor cells [29]. In addition, the NLRP3 inflammasome acts a negative modulator of tumorigenesis in colitis-associated cancer 2 via the regulation of intestinal homeostasis [30]. On the other hand, the protumor effect of the NLRP3 inflammasome in breast cancer progression has been described as resulting in infiltrating myeloid-derived suppressor cells and tumor-associated macrophages to the tumor site [31]. In addition, NLRP3 in HCC tissues has low expression in comparison with noncancerous tissues (such as cirrhosis), but it is significantly higher than normal tissues [32,33]. Thus, although the NLRP3 inflammasome plays an important role in various immune cells, including macrophages, it also might be expressed in human cancers and be implicated in tumor development and metastasis [34].

NK cells are innate lymphocytes that have a cytotoxic effect on cancer or stressed cells. Emerging evidence has shown that NK cells are critical for escaping cancer immunosurveillance, which is implicated in metastasis. Clinical data have shown that breast cancer, pancreatic cancer, and prostate cancer are not good for prognosis, because of the failure of NK immunosurveillance [26,35,36]. NK cells comprise a large proportion of liver lymphocytes but have shown a low frequency in patients with HCC. Moreover, the function of NK cells in patients with HCC is a low cytotoxicity and production of interferon-γ [21]. Thus, NK cell-mediated cancer surveillance is important on cancer development and metastasis, in HCC especially. To date, although many studies have demonstrated the role of the NLRP inflammasome in human cancers, a role of NLRP3 in HCC and its effect on NK cells have not yet been clarified. This study found that NK cytotoxicity was significantly increased in the deficiency of NLRP3 in HCC as compared with NLRP3-expressing HCC (Figure 1B). Interestingly, the deficiency of NLRP3 in HCC generated a high expression of NKG2D on NK cells led to a cytotoxicity (Figure 2). NK cells have a function in the immunosurveillance of tumors by controlling the balance of their inhibitory and activating receptors. NK-activating receptors do not act at a normal state, and they become activated if host cells are damaged. Among the NK-activating receptors, the natural killer group 2 member D (NKG2D) receptor is a representative receptor that is involved in the cytotoxicity that recognizes ligands from cancers [37]. However, various mechanisms inhibit the action of NKG2D receptor/NKG2DL to enable the immune escape of tumor cells. In fact, NKG2D-positive NK cells occur at a low frequency in various cancers, including pancreatic, gastric, and colorectal cancer [38], which leads to poor prognosis. Major histocompatibility complex class I chain-related protein A/B interacts with NK-activating receptors but is shed on many solid tumors [39,40]. A previous clinical study showed that because soluble MICA, which is shed on the surface of cancer cells, occurs at a high level in the serum of patients of various cancers, it is indicative of poor patient survival [41]. In response to increased expression of NKG2D on NK cells co-cultured with NLRP3 KO HCC, as expected, MICA on the surface of HCC cells was high expressed (Figure 3). These results assert that an ablation of NLRP3 in HCC cells enhance NK cytotoxicity through an increased interaction of NKG2D and MICA/B.

MMPs are a group of endopeptidases occurring in humans that are associated with the degradation of the extracellular matrix. This degradation causes tumor progression, biological functions, and an immunomodulatory response. Various studies have shown that the shedding of MICA on the surface of tumor cells is increased by proteases, including metalloproteinases (MMPs) and a disintegrin and metalloproteases (ADAMs) [42]. For instance, the overexpression of MMP-2 on the surface of renal cell carcinoma and membrane-type MMP-14 activity directly led to MICA shedding [43,44]. In addition, the proteolytic activity of MMP-9 in osteosarcoma induced the cleavage of MICA on the surface of cancer cells [24]. Thus, we examined whether a block of MICA shedding through a deficiency of NLRP3 in HCC was implicated in the expression of MMPs such as MMP2, MMP9, and MMP14. The expression of MMP2, MMP9, and MMP14 in NLRP3 KO HCC cells was significantly increased as compared with NLRP3 WT HCC cells (Figure 3 and Figure 5). The results implied that NLRP3 related to the activation of MMPs in HCC which led to MICA shedding. So far, nothing has known that NLRP3 inflammasome is implicated in led to shedding of MICA through the activation of MMPs. However, a few studies have shown that inflammasome pathway in molecular mechanisms of fibrosis involves the activation of MMPs through downstream IL-1R/MyD88 signaling [45,46].

Taken together, NK cytotoxicity was enhanced through a knockout of NLRP3 in HCC cells, and this was associated with NKG2D-MICA interaction. The shedding of MICA on the surface of HCC cells was inhibited by the lowered expression of MMP-2, MMP-9, and MMP-14 when is knocked NLRP3 out. Furthermore, in vivo experiment using xenograft mouse model, the NLRP3-deficient HCC cells delayed the tumor development and metastasis as well as interestingly enhanced a sensitivity of NK immunosurveillance in mice. Because all results support that a deletion of NLRP3 in HCC inhibits their development and metastasis through the control of their mechanism and an induction of NK cytotoxicity, we thus decide that to target NLRP3 in human HCC is good to NK cell immunotherapy. However, it might be equivalent only to human HCC SK-Hep1 Luc cells, in which NLRP3 is expressed.

## 4. Materials and Methods

### 4.1. Cell Lines and Culture

The NK cell line NK-92 was purchased from American Type Culture Collection (ATCC, Manassas, VA, USA), and the human HCC SK-Hep1-Luc cell line was provided by Professor Kuroda (Osaka University). NK-92 cells were cultured in alpha-MEM (Gibco, Grand Island, NY, USA) with 20% fetal bovine serum (FBS; Gibco), 100 U/mL penicillin and streptomycin (Gibco), 0.1 mM 2-mercaptoethanol (Sigma Aldrich, St. Louis, MO, USA), and rhIL-2 (200 U/mL, BioLegend, San Diego, CA, USA). SK-Hep1-Luc cells were cultured in Minimum Essential Media (GenDEPOT, Katy, TX, USA) supplied with 10% FBS (Gibco) and 1% penicillin and streptomycin (Gibco). Both were maintained at 37 °C in a humidified atmosphere with 5% CO_2_.

### 4.2. NLRP3 Knockout in HCC Using Lentiviral CRISPR Lenti-CRISPR-NLRP3/Cas9

Lentiviral CRISPR Lenti-CRISPR-NLRP3/Cas9 was provided by Kang’s Laboratory, Kyungpook National University (Daegu, South Korea). To edit the NLRP3 in SK-Hep1-Luc, the cells were incubated at a density of 5 × 10^6^ cells in a six-well bottom plate overnight, transfected with lentiviral supernatants of CRISPR-NLRP3 or CRISPR-control for one day, changed with fresh media with antibiotics for two days, and selected in 0.25 μg/mL puromycin for three days (Gibco, NY, USA).

### 4.3. RT-PCR and Quantitative RT-PCR Analysis

mRNA expression was analyzed by reverse transcriptase polymerase chain reaction (RT-PCR) and real-time quantitative PCR. Briefly, total RNA was isolated by TaKaRa MiniBest Universal RNA Extraction Kit (#9767, TaKaRa, Japan), and cDNA synthesis was performed using the PrimeScript 1st Strand cDNA Synthesis Kit (#6110A, TaKaRa) in accordance with the manufacturer’s instructions. Quantitative PCR was performed using SYBR Green (BioLine London, UK). Primer sequences were as follows: NLRP3: F 5′-AAAAGACTCATCCGTGTGCC-3′, R 5′-TTCCTGGCATATCACAGTGG-3′; GAPDH: F 5′-CACACCATCTTCCAGGAGC-3′, R5′-CATGAGTCCTTCCACGATACC-3′. The conditions for RT-PCR and quantitative PCR were as follows: 20 s at 94 °C for denaturation, 20 s at 60 °C for annealing, and 1 min at 72 °C for extension for 40 cycles quantitative RT-PCR was also processed for the melting cycle.

### 4.4. NK Cytotoxicity to Target Cells by LDH-Release Assay

The cytotoxic ability of NK-92 cells toward the target cells was analyzed using the CytoTox96 Non-Radioactive Cytotoxicity Assay Kit (Promega, Madison, WI, USA). Concisely, SK-Hep1-Luc cells transfected with Lenti-CRISPR-Control or CRISPR-NLRP3 were incubated at a density of 5 × 10^3^ cells in each well in 96-well bottom plates for 24 h and were then co-cultured with NK-92 cells of different densities (a ratio of E:T = 1:1, 2:1, 5:1, and 10:1) for 6 h. The supernatants were then harvested, transferred to each well of a fresh plate, and incubated in CytoTox96 reagent for 30 min. The absorbance was measured at 490 nm within 1 h after adding a stop solution using a microplate reader (BMG Labtech, Ortenberg, Germany).

### 4.5. Flow Cytometry Analysis

Briefly, target cells were incubated in six-well bottom plates for 24 h and then co-cultured at a ratio of 1:2 (target:effector) of NK-92 cells for 6 h. Then, NK-92 cells were harvested and stained with anti-NKG2D-APC, anti-NKp30-PE (eBioscience, San Diego, CA, USA) and VP (BD Bioscience, San Diego, CA, USA) for 30 min. After staining, cells were assessed using flow cytometry (Novocyte Flow Cytometer, ACEA Biosciences, San Diego, CA, USA). MICA/B on the surface of HCC cells was determined by staining with anti-MICA/B-PE (BD). The positive population of cells to each antibody was compensated by comparison of each emissive value.

### 4.6. Western Blot Analysis

To analyze protein expression within cells or tissues, all specimens were lysed by protein extraction buffer (Intron, Seoul, South Korea). The extracted proteins were quantified using the Bradford (Coomassie blue) assay (Gendepot, Katy, TX, USA) and then separated by electrophoresis, transferred to polyvinylidene fluoride microporous membrane (Millipore, Burlington, MA, USA), and blotted with first and second antibodies. Anti-NLRP3 (Abcam, Cambridge, UK), anti-PI3K (Cell Signaling, Danvers, MA, USA), anti-(p)-NF-kB (Millipore), anti-RAC-1 (Millipore), anti-(p)-MEK1/2 (Cell Signaling), (p)-ERK (Cell Signaling), and anti-GAPDH (Santacruz Biotechnology, Dallas, TX, USA) were used as first antibodies. For visualization, membranes were soaked in an enhanced chemiluminescent detection solution and detected by Chemi-doc (Millipore).

### 4.7. In Vivo Experiment Using Xenograft Model NRG Mice

All animal experiments were performed in accordance with National Research Council’s Guide (IACUC, Korea) for the Care and Use of Laboratory Animals. A protocol for in vivo experiments was approved by the Animal Experiments Committee of Duksung Women’s University (permit No. 2020-003-003). All mice (NRG, female, 5 weeks old) were purchased from Raonbio Co. Ltd. (Seoul, South Korea) and maintained in specific pathogen free supplied with food and water under a 12-h day/12-h night environment at 23 to 27 °C. After a one-week adaptive period, mice were intravenously injected with SK-Hep1-Luc cells (1 × 10^6^ cells/mouse) and transfected with lentiviral CRISPR-Control or CRISPR-NLRP3 to tail of each mouse. After four days, mice were grouped and intraperitoneally injected with NK-92 cells (2 × 10^6^ cells/mouse) twice a week. To visualize the tumors, mice were intraperitoneally injected with luciferin (3 mg per each mouse) and measured by in vivo imaging system (VISQUE, Gyeonggi-do, South Korea) once per week for six weeks.

### 4.8. IHC Staining Analysis

Liver tissues of all mice were resected, frozen with O.C.T. (Frozen Section Compound, Leica, Hesse, Germany), and stored at −80 °C until the experiment. Briefly, each specimen was sliced to a thickness at 5 μm and plated on a microscope slide. Plated tissues were hydrated in 70% ethanol, added to 3% H_2_O_2_ diluted in methanol, and washed with tap water and phosphate-buffered saline (PBS). Tissues were then soaked with blocking buffer (10% bovine serum albumin, 0.05% Tween 20/PBS) and then incubated with first, second, and third antibodies. After incubation, the tissues were colorized by DAB (Vector Labs, Burlingame, CA, USA) and stained with hematoxylin as a nucleic dye. All pictures were captured using light microscopy. First antibodies were as follows; MMP-9 (Cell Signaling), MICA/B (BioLegend), and NKG2D (Novusbio, Littleton, CO, USA).

### 4.9. Statistical Analyses

All data were processed using Microsoft Excel, and the results were presented as means ± SD. The comparison of several means was performed by one-way or two-way analysis of variance followed by Fisher’s exact test. Differences among group with *p* values less than 0.05 were considered significant.

## 5. Conclusions

In conclusion, we found that the expression of NLRP3 in human HCC impaired the function of NK cell immunosurveillance through a lowered interaction of NKG2D and MICA. When NLRP3 in HCC knocked out, MICA on the surface of HCC cells could be highly expressed because of the inhibited expression of MMPs, and which led to the effective NK cytotoxicity. These results indicate that NLRP3 might be used as an indicator to induce effective NK immunosurveillance in NLRP3-presenting HCC.

## Data Availability

All the data are contained within the article.

## Supplementary Materials

The following are available online at https://www.mdpi.com/article/10.3390/ijms22179285/s1, Figure S1: Expression of NLRP3 in HCC SK-Hep1 Luc cells with or without a co-culture of NK-92 cells; Figure S2: The secretion of IFN-γ between HCC SK-Hep1 Luc cells and NK-92 cells; and Figure S3: Expression of NKp44 on NK-92 cells in a co-culture of HCC SK-Hep1 Luc cells.
