# Supplementary material for: NLRP3 Deficiency in Hepatocellular Carcinoma Enhances Surveillance of NK-92 through a Modulation of MICA/B
Source: Int J Mol Sci. 2021 Aug 27;22(17):9285. doi: 10.3390/ijms22179285 (PMC8430511; doi:10.3390/ijms22179285)
Supplement: Supplementary file 1 [file ijms-22-09285-s001.zip › ijms-1335785-supplementary.pdf]

International Journal of
Molecular Sciences

MDPI

Supplementary Materials

NLRP3 Deficiency in Hepatocellular Carcinoma Enhances Surveillance of NK-92 through a Modulation of MICA/B

Hwan Hee Lee [1,2], Dongoh Kim [2], Joohee Jung [1,2], Hyojeung Kang [3,*], and Hyosun Cho [1,2,*]

Supplementary figures

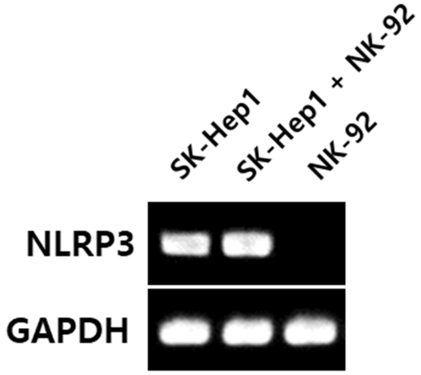

Figure S1. Expression of NLRP3 in HCC SK-Hep1 Luc cells with or without a coculture with NK-92.

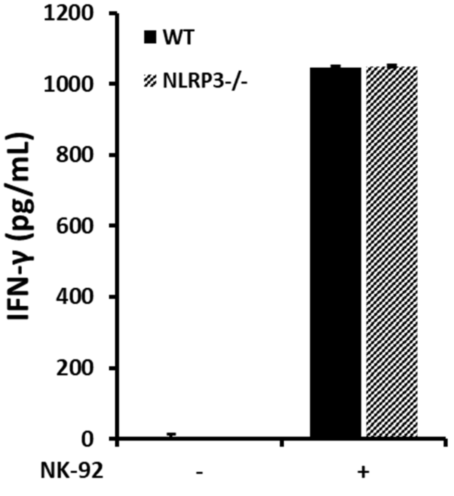

Figure S2. The secretion of IFN-γ between HCC SK-Hep1 Luc cells and NK-92 cells.

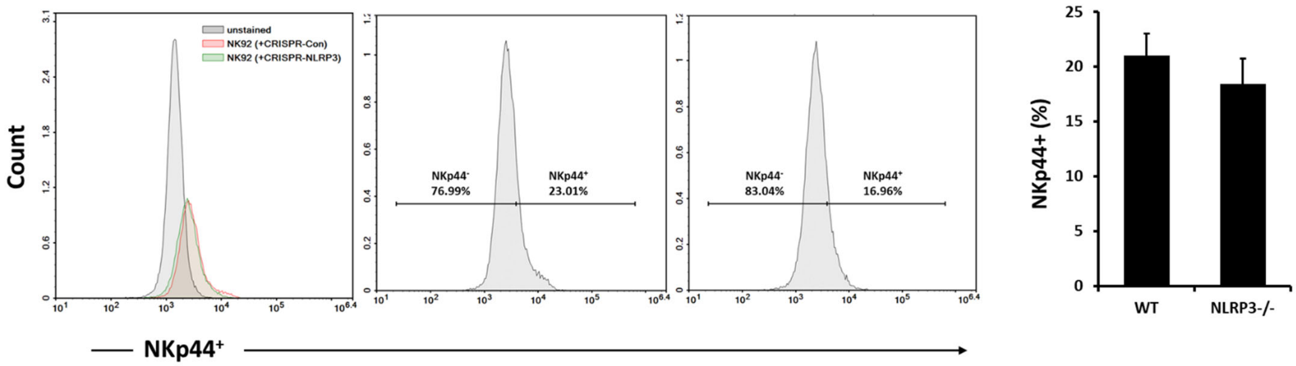

Figure S3. Expression of NKp44 on NK-92 cells in a co-culture of HCC SK-Hep1 Luc cells.
